# Book Notices

**Published:** 1870-10

**Authors:** 


### Book Notices.

The Practice of Medicine. By Thomas Hawkes Tanner, M.D., F. L. S., Member of the Royal College of Physicians, Fellow of the Royal Society of Literature, etc., etc. Fifth American, from the sixth London Edition, enlarged and thoroughly revised. Philadelphia: Lindsay & Blakiston. 1870.

This is an elegant volume of 1200 pages, containing a copious index, and numerous formula, besides a fair summary of the present state of Medical practice. It is a convenient work, but too well known to require an extended notice. In the appendix we find about forty pages of fine print, devoted to the consideration of climates for invalids, and mineral waters; which contain much valuable information.

For sale by S. C. Griggs & Co. Price, $6.00.

---

The Physical Exploration of the Rectum; with an Appendix on the Ligation of Hemorrhoidal Tumors. By William Bodenhamer, A.M., M.D. Illustrated by numerous drawings. New York: Wm. Wood & Co., 61 Walker St. 1870.

This is a monograph of 54 pages, on a subject of much practical importance. It is illustrated by numerous well-executed cuts, and written in good style. The author has studied his subject well, to which has been added an ample practical experience. Every practitioner will find it very useful and convenient.

---

A Guide to the Examination of Urine; for the Practitioner and Student. By J. Wickham Legg, M.D., Member of the Royal College of Physicians; Physician to the St. George, Hanover Square, Dispensary. Second Edition. Philadelphia: Lindsay & Blakiston. 1870.

This is a small volume of 88 pages in flexible binding, and contains a plain, concise account of the most practicable methods of examining the Urine, for the purpose of ascertaining its qualities and composition, in health and in disease. Its cost is trifling, and it will be found very convenient both for students and practitioners.

---

Half-Yearly Compendium of Medical Science. Part 5, July, 1870. Philadelphia: S. W. Butler, M.D., 115 South Seventh Street.

This is a copious abstract of the more important matter that is published in American Medical Journals, with some additional from foreign sources. The present number is printed on good paper, and contains 304 pages. For such practitioners as are able to subscribe for only one or two regular medical periodicals, the Compendium is a most valuable publication. It is edited by Drs. S. W. Butler, D. C. Brinton, and G. H. Naphys, all industrious men. Price, $3.00 per annum.

